# Development of a New Tacaribe Arenavirus Infection Model and Its Use to Explore Antiviral Activity of a Novel Aristeromycin Analog

**DOI:** 10.1371/journal.pone.0012760

**Published:** 2010-09-16

**Authors:** Brian B. Gowen, Min-Hui Wong, Deanna Larson, Wei Ye, Kie-Hoon Jung, Eric J. Sefing, Ramona Skirpstunas, Donald F. Smee, John D. Morrey, Stewart W. Schneller

**Affiliations:** 1 Department of Animal, Dairy, and Veterinary Sciences, Institute for Antiviral Research, Utah State University, Logan, Utah, United States of America; 2 Molette Laboratory for Drug Discovery Research, Chemistry and Biochemistry Department, Auburn University, Auburn, Alabama, United States of America; 3 Department of Agriculture and Food, State of Utah, Logan, Utah, United States of America; University of California San Francisco, United States of America

## Abstract

**Background:**

A growing number of arenaviruses can cause a devastating viral hemorrhagic fever (VHF) syndrome. They pose a public health threat as emerging viruses and because of their potential use as bioterror agents. All of the highly pathogenic New World arenaviruses (NWA) phylogenetically segregate into clade B and require maximum biosafety containment facilities for their study. Tacaribe virus (TCRV) is a nonpathogenic member of clade B that is closely related to the VHF arenaviruses at the amino acid level. Despite this relatedness, TCRV lacks the ability to antagonize the host interferon (IFN) response, which likely contributes to its inability to cause disease in animals other than newborn mice.

**Methodology/Principal Findings:**

Here we describe a new mouse model based on TCRV challenge of AG129 IFN-α/β and -γ receptor-deficient mice. Titration of the virus by intraperitoneal (i.p.) challenge of AG129 mice resulted in an LD_50_ of ∼100 fifty percent cell culture infectious doses. Virus replication was evident in the serum, liver, lung, spleen, and brain 4–8 days after inoculation. MY-24, an aristeromycin derivative active against TCRV in cell culture at 0.9 µM, administered i.p. once daily for 7 days, offered highly significant (*P*<0.001) protection against mortality in the AG129 mouse TCRV infection model, without appreciably reducing viral burden. In contrast, in a hamster model of arenaviral hemorrhagic fever based on challenge with clade A Pichinde arenavirus, MY-24 did not offer significant protection against mortality.

**Conclusions/Significance:**

MY-24 is believed to act as an inhibitor of S-adenosyl-L-homocysteine hydrolase, but our findings suggest that it may ameliorate disease by blunting the effects of the host response that play a role in disease pathogenesis. The new AG129 mouse TCRV infection model provides a safe and cost-effective means to conduct early-stage pre-clinical evaluations of candidate antiviral therapies that target clade B arenaviruses.

## Introduction

Junín and other South American hemorrhagic fever-causing viruses pose a considerable public health threat as emerging infectious disease agents and because of their potential for intentional release [1]. All of the highly pathogenic New World arenaviruses (NWA; Junín, Machupo, Guanarito, Sabia), including the recently identified Chapare virus, phylogenetically segregate into clade B [2], and require maximum biosafety level 4 (BSL-4) containment facilities for their study. Presently, there are no clade B arenavirus infection models outside of newborn mice suitable for early stage antiviral drug development and proof-of-concept studies. There are several guinea pig and nonhuman primate models based on infection with authentic BSL-4 arenaviral hemorrhagic fever agents, but they are not readily available to most researchers [3]. Moreover, studies in BSL-4 containment and with larger animal species are cost-prohibitive for use in early pre-clinical drug development.

Tacaribe virus (TCRV) is a nonpathogenic member of clade B that is ∼70% identical to Junín virus (JUNV) at the amino acid level [4]. However, despite its relatedness to the highly pathogenic NWA, TCRV lacks the ability to antagonize the host interferon (IFN) response [5], which likely contributes greatly to its inability to cause disease in mature animals. Because of the apathogenicity of TCRV in mice and other rodents, a newborn mouse model was established to evaluate lead antiviral compounds *in vivo*, primarily with the intent to demonstrate proof-of-concept in a Clade B NWA model [4], [6]. Due to the many challenges of working with newborn mice and their underdeveloped cellular and immune response to infectious agents, an alternative model to study clade B arenavirus infection biology and evaluate candidate therapies is needed.

AG129 IFN-α/β and -γ receptor knockout (KO) mice were originally described to have increased susceptibility to severe infection with the prototypical arenavirus, lymphocytic choriomeningitis virus (LCMV), as well as vaccinia virus [7]. Recently, the AG129 mice were used to develop a dengue hemorrhagic fever disease model that manifests vascular leak, thus, more closely resembling the human condition [8]. In a study investigating the contributions of type I and II IFN antiviral responses to Sindbis virus infection, wild-type and IFN-γ receptor KO (G129) mice were found to be resistant to challenge, whereas the IFN-α/β receptor KO (A129) and the double KO AG129 mice succumbed to infection [9]. Importantly, the AG129 mice produced a lethal viral hemorrhagic fever (VHF)-like disease not observed in the Sindbis virus-infected A129 mice. Because TCRV does not disrupt IFN production [5], the AG129 mice may provide fertile ground for viral replication that culminates in a lethal viscerotropic disease, thereby providing a model that can be used to evaluate antiviral drug candidates for the treatment of acute arenaviral infections.

To date, there are limited options for treating JUNV infection in cases of Argentine hemorrhagic fever. Immune plasma has been reported to be effective at reducing case-fatality rates when administered within a week from the onset of illness [10]. In a clinical trial with limited enrollment, ribavirin therapy had an antiviral effect on several measured disease parameters including viral load and delay in time of death in patients who succumbed [11]. Notably, however, both immune plasma and ribavirin treatments have been associated with neurologic sequelae following the resolution of the acute phase of the disease [12]. The use of ribavirin has also been explored in several cases of Bolivian hemorrhagic fever [13], but larger numbers of patients are needed to convincingly demonstrate efficacy. Finally, in a landmark study wherein ribavirin was used to treat sever cases of Lassa fever, significant efficacy was demonstrated [14]. Notably, however, ribavirin lacks specificity [15], is associated with considerable toxicity [16], and is not approved by the FDA for the indication of treating any form of arenaviral hemorrhagic fever [1].

There are several new antiviral drug candidates that have demonstrated efficacy in small animal models of acute NWA infection. Favipiravir (T-705) has been shown to be highly effective in the hamster Pichinde virus (PICV) model of arenaviral hemorrhagic fever [17], and is capable of treating advanced disease [18]. Presumably, the mode of antiviral action in arenaviruses is through inhibition of the viral polymerase, as has been shown for influenza virus [19]. ST-294, a potent inhibitor of NWA membrane fusion has also demonstrated activity in a newborn mouse TCRV infection model [4].

Several biologics that enhance the host antiviral response are also being considered. Although Lassa fever is thought to be resistant to the effects of type I IFN [20], a recent study demonstrated sensitivity of several strains Lassa virus to IFN-α and -γ in cell culture [21]. Moreover, treatment with consensus IFN-α, alone or in combination with ribavirin has proven effective in the hamster PICV infection model [22], [23]. A novel therapy based on the targeting of anionic phospholipids exposed on infected cells and virions has shown promise in studies employing a guinea pig model based on infection with an adapted PICV [24]. Despite the present efforts to develop therapies for the treatment of arenaviral hemorrhagic fevers, most are in the early stages of development, and new classes of inhibitors will most certainly be needed.

**Figure 1:**
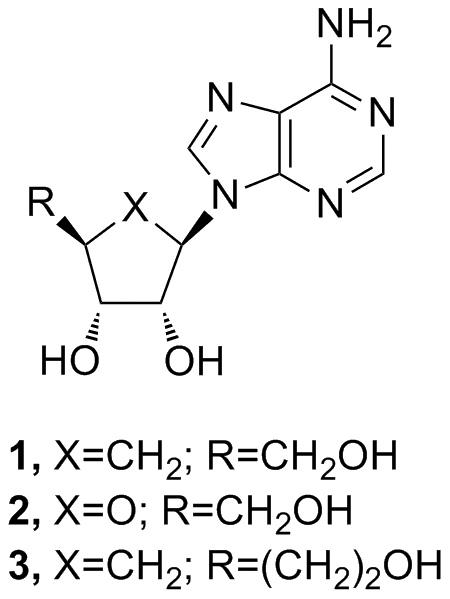
Chemical structure of MY-24 and derivatives. Aristeromycin (**1**), adenosine (**2**), 5′-homoaristeromycin (MY-24)(**3**).

**Figure 2:**
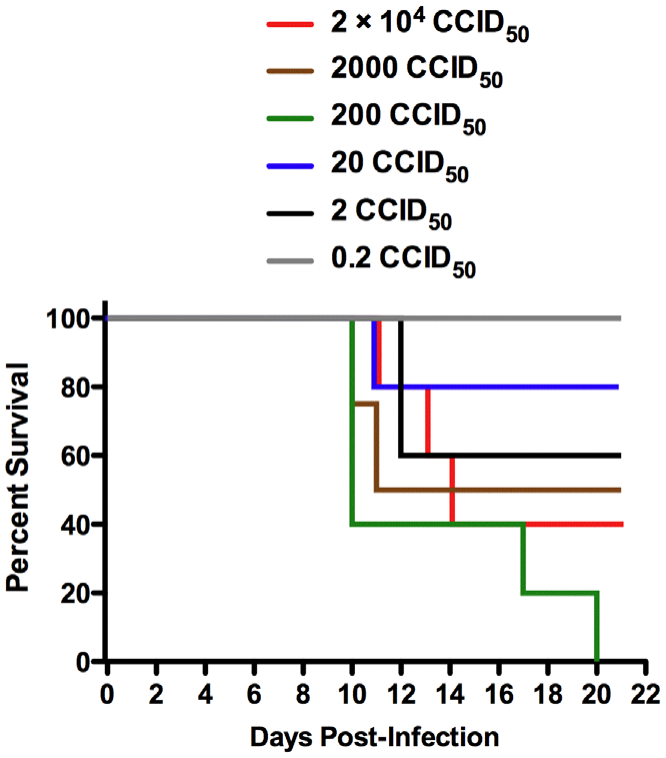
Survival of AG129 mice challenged with TCRV. Groups of 5 mice/group were inoculated with the indicated CCID_50_ dose of TCRV. Mortality was monitored over a 21-day period. Percent survival for the 2,000 CCID_50_ group was based on 4 animals due to the loss of one animal on day 2 from causes not believed to be related to TCRV infection.

**Figure 3:**
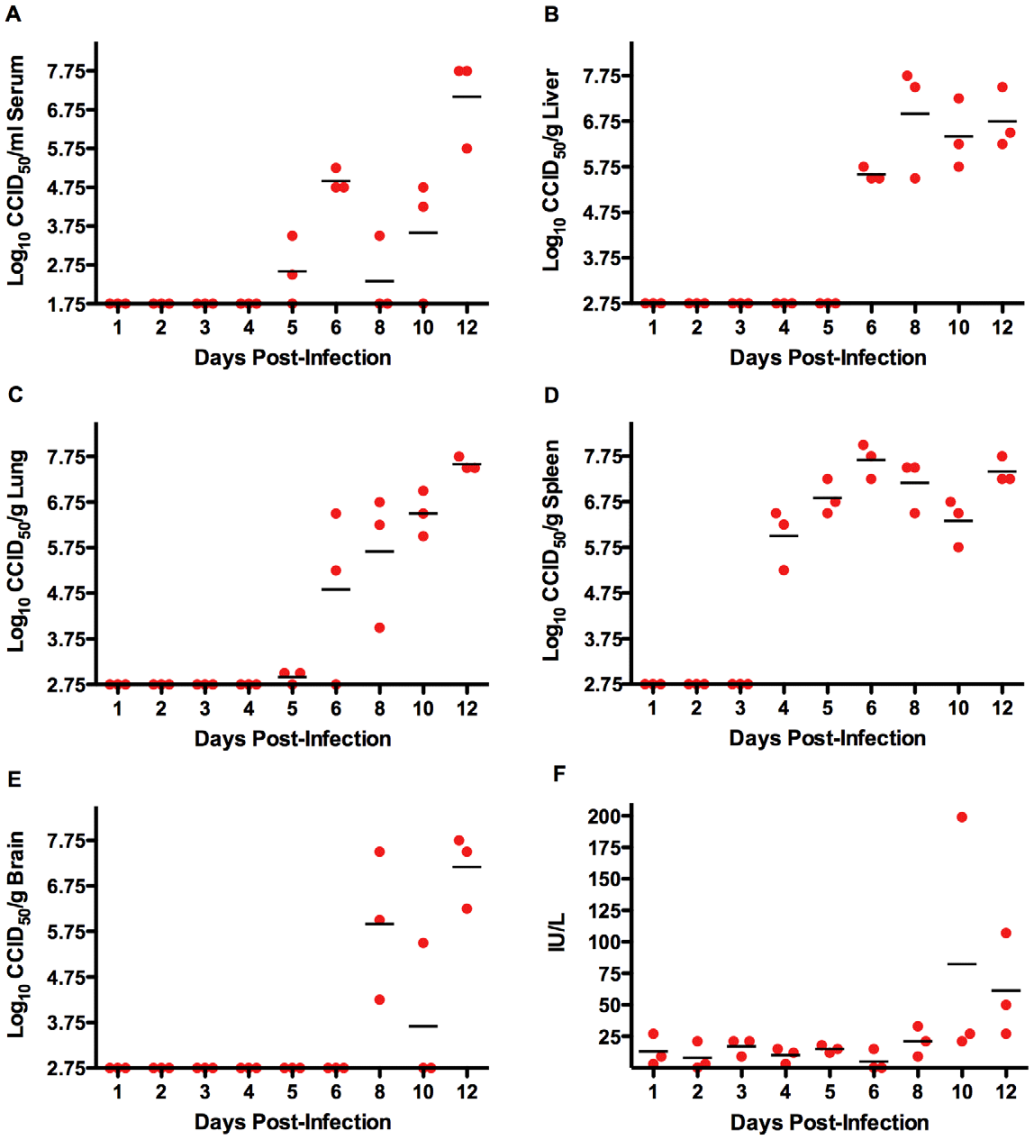
Time course analysis of tissue TCRV titers and ALT levels in AG129 mice. Groups of 3 animals were sacrificed on the specified days during infection for analysis of A) serum, B) liver, C) lung, D) spleen, and E) brain virus titers, and F) serum ALT concentration. The day-12 group started with 6 animals, with 3 succumbing prior to time of sacrifice.

Carbocyclic nucleosides have provided a foundation for discovering new biological agents, including antivirals [25], [26]. Among this class of compounds, aristeromycin (Figure 1, **1**), which is the naturally occurring carbocylic nucleoside analog of adenosine (Figure 1, **2**) [27], has been particularly valuable in the search for new antivirals because of its inhibition of host cell *S*-adenosylhomocysteine hydrolases [28], an enzyme that plays a role in metabolic methylations requiring *S*-adenosylmethionine as enzymatic cofactor [29]. However, the potential of aristeromycin is limited by its ready intracellular conversion to the 5′-nucleotides that renders it toxic [30], [31], [32], [33]. As part of a study to circumvent this toxicity, 5′-homoaristeromycin (Figure 1, **3**) was reported to have activity against vaccinia, cowpox, and monkeypox viruses [34]. In the following, we report the details of our synthesis of 5′-homoaristeromycin, referred to herein as MY-24, and its evaluation in a newly developed AG129 mouse TCRV infection model.

## Materials and Methods

### Ethics statement

All animal procedures complied with USDA guidelines and were conducted at the AAALAC-accredited Laboratory Animal Research Center at Utah State University under protocols 1229 and 1425, approved by the Utah State University Institutional Animal Care and Use Committee.

### Animals

Four to seven week-old AG129 IFN-α/β and -γ receptor-deficient mice were obtained from Dr. Justin Julander's breeding colony at Utah State University. They were fed irradiated mouse chow and autoclaved water *ad libitum*. Female 60–100 g golden Syrian hamsters were obtained from Charles River Laboratories (Wilmington, MA) and acclimated for a minimum of 6 days prior to experimentation. They were fed standard hamster chow and tap water *ad libitum*.

### Viruses

TCRV, strain TRVL 11573, was obtained from American Type Culture Collection (ATCC; Manassas, VA). The virus stock (2 passages in Vero 76 African green monkey kidney cells) used was from a clarified cell culture lysate preparation concentrated using an Amicon stirred ultrafiltration cell (Millipore, Danvers, MA). The JUNV vaccine strain, Candid 1, was provided by Dr. Robert Tesh (World Reference Center for Emerging Viruses and Arboviruses, University of Texas Medical Branch, Galveston, TX). PICV, strain An 4763, was provided by Dr. David Gangemi (Clemson University, Clemson, South Carolina). The virus was passaged once through hamsters and once in Vero (African green monkey kidney) cells. PICV stocks for challenge efficacy studies were prepared from pooled livers harvested from infected hamsters. Stocks for cell culture studies were from clarified Vero cell culture lysates. JUNV was amplified in Vero cells and the virus stock was prepared from clarified cell culture lysates.

### Compounds

The synthesis of MY-24 has previously been reported in communication form [34]. However, the experimental details have not been described. Due to the biological potential of MY-24, the detailed procedures are provided as supporting methodology (Figure S1 and Text S1). Ribavirin was supplied by ICN Pharmaceuticals, Inc. (Costa Mesa, CA). For *in vivo* studies, both MY-24 and ribavirin were dissolved in sterile saline solution and administered by intraperitoneal (i.p.) injection.

### Cell culture antiviral assays

Vero and Vero 76 cells were obtained from ATCC and maintained in minimal essential medium (MEM) supplemented with 0.18% NaHCO_3_ and 10% fetal bovine serum (FBS; Hyclone Thermo Scientific, Logan, UT). Cell cultures in 96-well microtiter plates were ∼80% confluent at time of infection with 5 cell culture 50% infectious doses (CCID_50_) of JUNV, PICV, or TCRV prepared in MEM containing 2% FBS. Varying concentrations of MY-24 and ribavirin (positive control) solubilized in MEM were added to test wells at the time of infection. To determine cell cytotoxicity, compounds were added to cultures devoid of virus infection. Plates were incubated at 37°C, 5% CO_2_, until virus-infected mock-treated control wells were observed to have maximal viral cytopathic effect CPE (∼7 to 8 days), at which time cell viability was determined by neutral red (NR) dye uptake as previously described [17]. The mean effective concentration (EC_50_) of each compound and the concentration that reduced cell viability by 50% (CC_50_) were determined by regression analysis. Virus yield reduction (VYR) experiments were conducted to determine the effect of MY-24 on infectious virus. Concentrations of compound that reduce virus yield by 1 log_10_ (EC_90_) were determined by regression analysis. Selectivity index (SI) values were calculated as the CC_50_/EC_50_ for the CPE reduction (CPER) NR-based assays, and as CC_50_/EC_90_ for the VYR assays.

### TCRV AG129 mouse model development and challenge efficacy studies

For all studies mice were age and gender matched so that the group compositions would be similar within experiments. In the initial experiments, weights were not measured to limit handling and exposure of the immunocompromised AG129 mice. For the titration study, mice in each group were challenged by intraperitoneal (i.p.) injection with varying CCID_50_ of TCRV spanning 6 orders of magnitude and observed for 21 days. Because several of the mice continued to appear ill on day 21, we collected liver, spleen, brain, and serum for virus titer determination, as described below.

A longitudinal analysis of viral titers and ALT levels was performed by sorting mice into groups of 3 to 6 animals and challenging them with ∼200 CCID_50_ of TCRV. The mice were observed for 1 to 12 days, and sacrificed on days 1–6, 8, 10, and 12. The day-12 group had 6 animals in anticipation of several animals succumbing prior to time of sacrifice. Serum was assayed for viral burden and ALT levels. Tissues were collected for liver, lung, spleen, and brain virus titer determination, as described below. Histopathology was also determined at various times during the course of infection. Tissue sections were fixed in formalin and sent to the Utah Veterinary Diagnostic Laboratory (Logan, UT) for histological examination.

In the TCRV challenge MY-24 efficacy experiments, mice were sorted into groups of 10 to 15 animals for drug treatment groups and 15 to 25 for the placebo groups. MY-24 treatments were administered starting 4 h prior or 1, 3 or 5 days after challenge with 200 CCID_50_ of TCRV. Animals were treated i.p. once daily for 7 days with 25 to 150 mg/kg/day of MY-24, 50 mg/kg/day ribavirin, or saline placebo. In one of the experiments, 5 mice from each group were sacrificed on day 8 of infection. Serum, liver, lung, spleen, and brain samples were collected for assaying virus titers as described below. The mice were observed for 3 to 4 weeks for signs of morbidity and mortality. Three to six sham-infected mice were included as normal controls for the infections. A subset of uninfected animals treated with 75 or 150 mg/kg/day of MY-24 was also included in the first experiment to assess possible toxicity.

### Hamster PICV challenge MY-24 efficacy studies

Hamsters were weighed on the morning of treatment and grouped so that the average hamster weight per cage across the entire experiment varied by less than 5 grams. Animals were treated as indicated with 5 to 100 mg/kg/day doses of MY-24 or vehicle placebo 4 h prior to i.p. challenge with ∼2 plaque-forming units (PFU) of PICV. Ribavirin (40 mg/kg/day) was included as a positive control and given by the same route and following the same schedule. Five hamsters from each group (up to 10 for the placebo groups) were sacrificed on day 7 of infection and sera were collected for assaying alanine aminotransferase activity and virus titers were determined for both liver and serum samples as described below. The remaining 10 animals (20 for the placebo group) were observed 21 days for mortality. Three to four sham-infected controls were included for comparison to establish baselines for all test parameters. In separate studies conducted in uninfected hamsters, tolerability of doses up to 100 mg/kg/day of MY-24 was evaluated prior to challenge efficacy experiments.

### Tissue virus titer determinations

Virus titers were assayed using an infectious cell culture assay as previously described [17]. Briefly, a specific volume of tissue homogenate or serum was serially diluted and added to triplicate wells of Vero 76 cell monolayers in 96-well microplates. The viral CPE was determined 7–8 days post-virus inoculation and the 50% endpoints were calculated as described [35]. The assay detection ranges were 2.8–9.5 log_10_ CCID_50_/g of tissue or 1.8–8.5 log_10_ CCID_50_/ml of serum. In samples presenting with undetectable tissue or serum virus, a value of <2.8 or 1.8 log_10_ was assigned, respectively. Conversely, in cases wherein virus exceeded the detection range, a value of>9.5 or 8.5 log_10_ was assigned. For graphic representation and statistical analysis, respective values of 1.8, 2.8, 8.5, or 9.5 log_10_ were assigned as needed for samples with undetectable or saturated virus levels.

### Serum alanine aminotransferase (ALT) determinations

Detection of ALT in serum is an indirect method for evaluating liver damage. Per the manufacturer's recommendations, serum ALT levels were measured using the ALT (SGPT) Reagent Set purchased from Pointe Scientific, Inc. (Lincoln Park, MI). The reagent volumes were adjusted for analysis on 96-well microplates.

### Statistical analysis

Kaplan-Meier survival plots and all statistical evaluations were done using Prism (GraphPad Software, CA). The log-rank test was employed for survival analysis. For analyzing differences in viral titers and ALT levels, a one-way analysis of variance (ANOVA) with Newman-Keuls post test or the Kruskal-Wallis test with the Dunn's post test was performed based on Gaussian distribution of the data. The Mann-Whitney test (two-tailed) was used for comparing mean day of death.

## Results

### In vitro anti-arenavirus activity of MY-24

The activity of the aristeromycin derivative, MY-24, was investigated in several cell culture-based arenavirus infection model systems. As shown in Table 1, MY-24 demonstrated moderate activity against TCRV, JUNV, and PICV by VYR with EC_90_ values ranging from 0.9 to 2.4 µM. Notably, by CPER assay, the clade B arenaviruses (TCRV and JUNV) were found to be more sensitive to MY-24. Ribavirin, included as a positive control, was active in the range of 8–16 µM versus the same panel of arenaviruses. MY-24 had markedly lower CC_50_ values compared to ribavirin, resulting a 4-fold difference in VYR SI values ranging from 12 to 31 for MY-24 and 119 to 135 for ribavirin.

**Table 1:** *In vitro* inhibitory effects of MY-24 and ribavirin against arenaviruses^a^.

			MY-24^c^			Ribavirin c	
Virus	Assay b	CC_50_ ± SD	EC_50/90_ ± SD	SI d	CC_50_ ± SD	EC_50/90_ ± SD	SI d
TCRV	CPER	28±8.2	0.9±0.2	31	1106±356	12±2.5	92
	VYR		0.9±0.2	31		8.2±2.0	135
JUNV	CPER	33±5.7	2.4±1.2	14	1188±356	11±5.7	108
	VYR		1.1±0.6	30		10±3.9	119
PICV	CPER	28±15	16±10	2	1024±217	16±8.2	64
	VYR		2.4±1.2	12		7.8±2.5	132

a Data are the mean and standard deviations from 3 separate experiments in Vero (JUNV and PICV) or Vero 76 (TCRV) cells.

b Cytopathic effect reduction (CPER) based on neutral red dye uptake by viable cells; virus yield reduction (VYR).

c CC_50_ and EC_50_ values are inµM.

d Selectivity index (SI)  =  CC_50_/EC_50/90_.

### Characterization of TCRV infection in AG129 mice

Because MY-24 demonstrated better antiviral activity against the more medically relevant clade B arenaviruses in cell culture (Table 1), we pursued the development of a rodent model based on challenge with TCRV. We initially challenged weanling hamsters i.p. with up to 10^6^ CCID_50_ of TCRV and found them to be refractory to infection, with no apparent signs of illness or weight loss (data not shown). We next explored TCRV model development in AG129 IFN-α/β and -γ receptor-deficient mice. We hypothesized that devoid of the critical IFN antiviral response, these mice would be susceptible to productive TCRV infection. As shown in Figure 2, the AG129 mice were sensitive to the virus at varying degrees based on the viral inoculum, with disease progressing slowly and animals first succumbing on day 10 of infection.

Because some of the surviving mice continued to show varying degrees of mild to moderate disease signs (lethargy, ruffled fur, and hunched posture) towards the final days of the 3 week study, we sacrificed all surviving animals on day 21 to measure systemic viral burden and tissue titers. In the 4 surviving animals that were challenged with 2×10^3^ or greater CCID_50_, all had 5 to 6 log_10_ of virus in the brain and spleen, 2 of 4 had 5 log_10_ of liver virus, and 3 of 4 had 3.5 to 5 log_10_ of serum virus (data not shown). Only ∼20% of the samples collected from the surviving mice challenged with 20 or 2 CCID_50_ of TCRV had detectable levels of virus on day 21.

A follow-up study was conducted to characterize the progression of TCRV infection during the acute phase of disease in AG129 mice. As seen in Figure 3A–E, all tissues examined harbored virus. The first organ to have significant amounts of TCRV replication was the spleen, with ∼6 log_10_ CCID_50_/g on day 4 of the infection, and sustained virus burden out to day 12 (Figure 3D). In several spleen samples collected on and after day 8, white pustule-like spots and a pale light color were evident by gross visual examination. Virus first became apparent systemically on day 5 in 2 of the 3 mice, with sporadic titers through day 10, and a spike up to ∼7 log_10_ CCID_50_/ml detected on day 12 (Figure 3A). Remarkably, liver virus titer went from undetectable on day 5 to ∼6–7 log_10_ CCID_50_/g on days 6–12 (Figure 3B). A slight hint of lung virus could be detected as early as day 5, with a substantial incremental increase thereafter of approximately 1 log_10_/day as disease progressed (Figure 3C). TCRV was found in the brain in 7 of 9 animals on day 8 and later (Figure 3E). It is possible that virus was present as early as day 7 since we did not include that time point in the analysis. Overall, ALT levels were fairly normal despite considerable viral burden; however, a few animals did show some elevation on days 10 and 12 (Figure 3F). Considering that the mean day of death in animals that succumb from TCRV infection is ∼12 days, with a range of 10–20 days, it is not surprising to see peak infectious TCRV loads at day 12. Notably, the day-12 analysis is likely an underestimate of the viral burden since the 3 sickest animals had succumbed prior to the time of sample collection.

**Figure 4:**
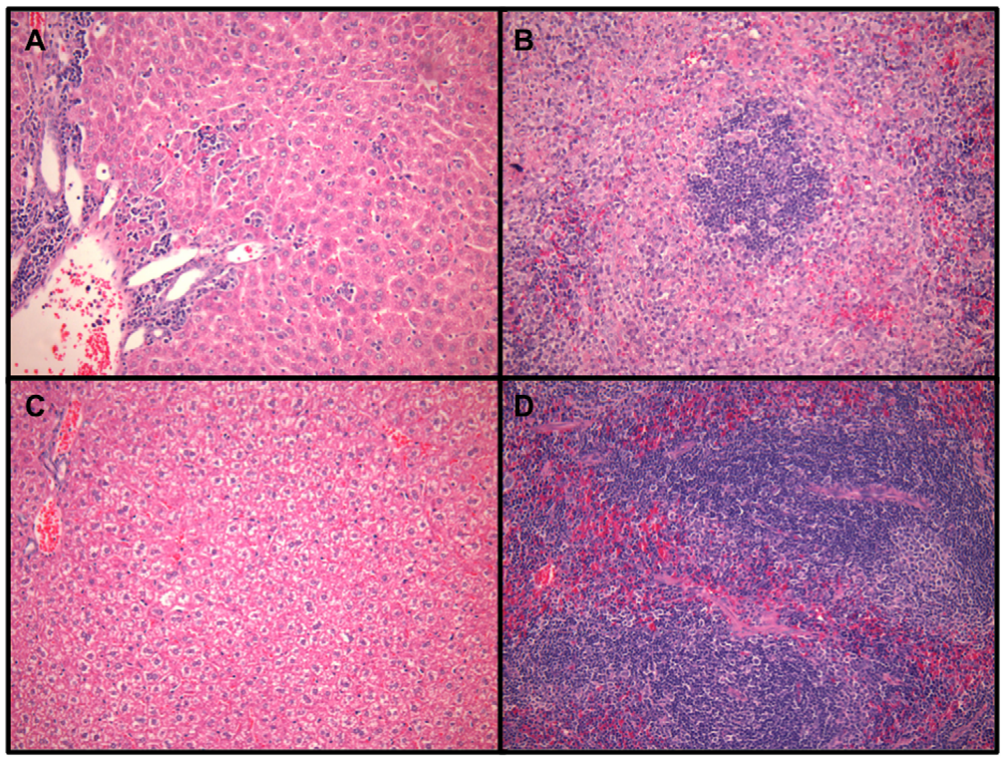
Histologic examination of liver and spleen sections from TCRV-infected AG129 mice. Representative A) liver and B) spleen histopathology on day 8 of TCRV infection in AG129 mice. C) Liver and D) spleen tissue from healthy sham-infected mice. Tissues were stained with hematoxylin and eosin.

Obvious evidence of disease was not histologically observed until day 8 of infection. Typical liver lesions included moderate numbers of portal lymphocytes and histiocytes (Figure 4A) and scattered degenerate/necrotic hepatocytes surrounded by small numbers of neutrophils or lymphocytes. Spleens of infected mice had hyperplastic follicles, follicular lympholysis and increased numbers of interstitial neutrophils (Figure 4B). Findings from day-10 livers and spleens had similar pathology to that described for day-8 tissue samples (data not shown). There was no kidney or brain pathology associated with advanced TCRV infection in the AG129 mice. However, there was mild perivascular edema with small numbers of mixed inflammatory cells surrounding larger vessels in lungs of ∼25% of the animals on days 8 and 10 of infection (data not shown). Taken together, the virus titer and histological findings indicate that TCRV-induced disease progresses slowly in the AG129 mice, providing an ample window of therapeutic opportunity to assess anti-arenaviral drug candidates in a murine system.

### Evaluation of MY-24 prophylaxis in AG129 mice challenged with TCRV

**Figure 5:**
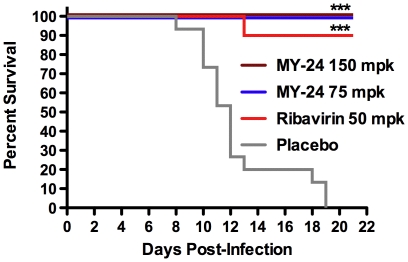
MY-24 protects AG129 mice challenged with TCRV from mortality. Mice were treated i.p. once daily for 7 days with the indicated mg/kg (mpk) doses of MY-24 or ribavirin. Treatment was initiated 4 hours prior to infection. Data shown for the high-dose MY-24, low-dose MY-24, ribavirin, and placebo groups are based on 9, 8, 9, and 15 animals per group, respectively. ***P<0.001 compared to saline placebo-treated animals.

Having gained an understanding of the natural history of disease in the AG129 mouse TCRV mouse infection model, we sought to evaluate MY-24 in the newly established model. MY-24 was dosed at 150 and 75 mg/kg/day i.p. for 7 days. We treated the mice only once daily to limit handling of the type I and type II IFN system-compromised AG129 mice. As demonstrated in Figure 5, MY-24 treatment regimens resulted in 100% protection against a lethal TCRV challenge dose. Ribavirin was also effective and protected 89% of the mice from mortality. Six uninfected mice treated in parallel with 150 mg/kg/day (n = 3) or 75 mg/kg/day (n = 3) of MY-24 all survived the treatment regimen without any signs of adverse effects.

Notably, we did observe that the infected animals treated with MY-24 had ruffled fur and were lethargic compared to the control animals towards the end of the study, which prompted us to weigh them on days 18 and 21. Average group weight gain of 1.9 (150 mg/kg/day dose) and 1.1 (75 mg/kg/day dose) grams over that period suggested to us that they were on their way to recovery. Some of the ribavirin-treated animals also presented with ruffled fur and one had left hind leg paralysis. These animals also gained weight (1 g) from days 18 to 21 and appeared to be in a state of recovery. For comparison, the sham-infected control mice (n = 5) gained 0.9 g over that 3-day period.

**Figure 6:**
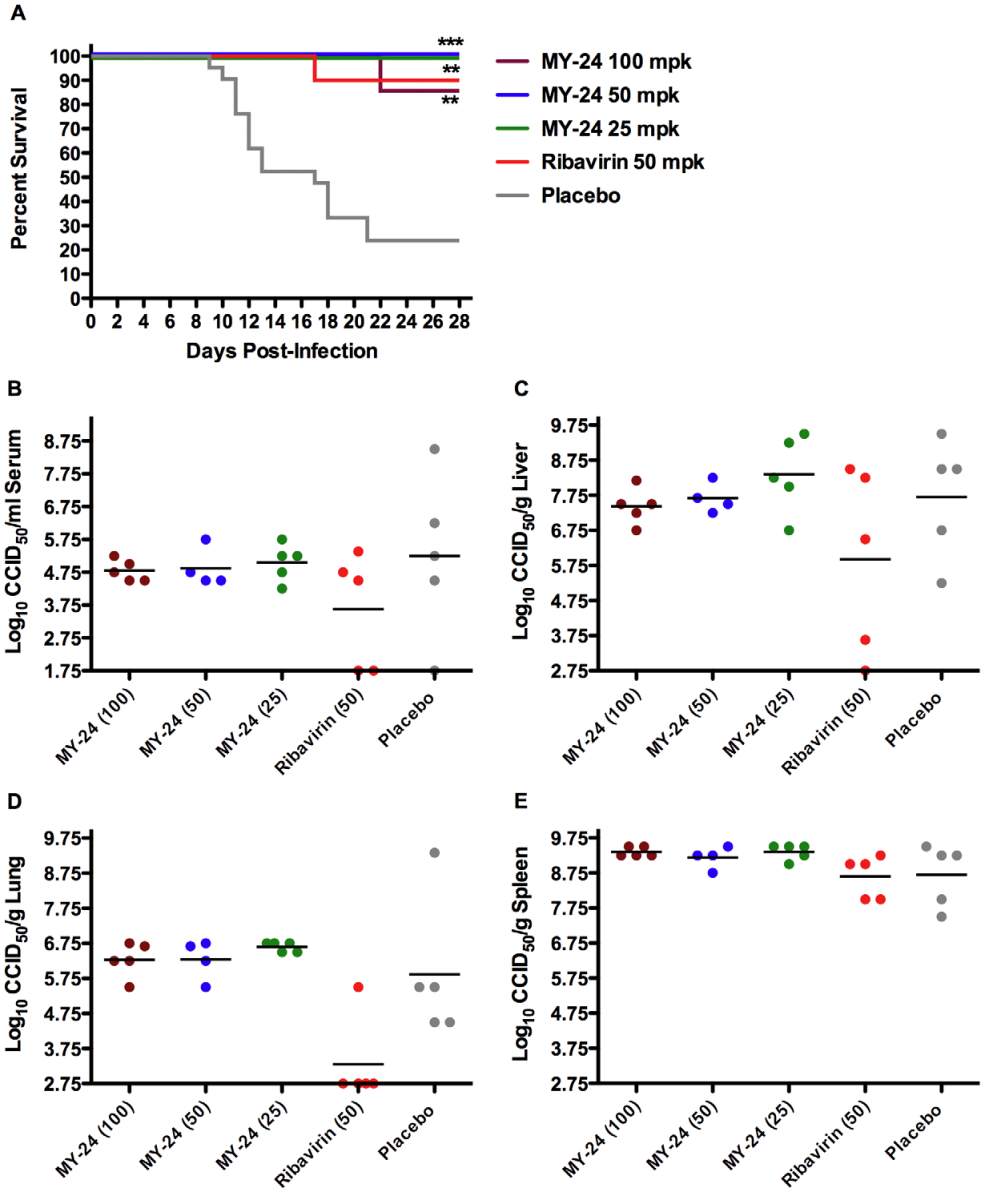
MY-24 protects AG129 mice against lethal TCRV infection despite lack of inhibition of viral replication. Mice were treated i.p. once daily for 7 days with the indicated doses of MY-24 or ribavirin, starting 4 h prior to TCRV challenge. A) Survival data are based on 7, 10, 10, 10, and 20 animals per group for the high-dose MY-24, intermediate dose MY-24, low-dose MY-24, ribavirin, and placebo groups, respectively. An additional 4 to 5 mice per group were sacrificed on day 8 of infection for B) serum, C) liver, D) lung, and E) spleen virus titer determinations. **P<0.01, ***P<0.001 compared to saline placebo-treated animals.

A follow-up evaluation of MY-24 in the AG129 mouse TCRV infection model was conducted to verify the results from the initial experiment (Figure 5) and to assess the impact of the compound on viral burden. Again, dramatic efficacy was observed in mice treated with MY-24 in the context of survival (Figure 6A). Complete protection was seen in the 25 and 50 mg/kg/day groups, with only a single mouse (out of a total of 7) succumbing in the 100-mg/kg/day group on day 22 of the infection, well after the mean day of death (13.7 days) of animals receiving placebo. Ribavirin protected 90% of challenged mice. By visual inspection of the animals during the study, several of the ribavirin treated animals presented with varying degrees of lethargy and ruffled fur starting on day 12 and thereafter. This was also apparent in some of the mice in the 100- and 50-mg/kg/day MY-24 groups on day 17 and beyond, but to a lesser degree in the 50-mg/kg/day group. Despite their ruffled appearance, most of these animals survived the 28-day observation period. More severe lethargy and ruffling of fur were observed in placebo-treated animals prior to succumbing from infection.

In addition to survival, day 8 viral loads from serum and liver, spleen, lung (Figure 6B–E), and brain tissues (data not shown) were also examined. Virus was present in all tissues except for brain. Lack of detectable virus may have been due to a slower development of brain virus titers in this particular experiment. MY-24 did not have any impact on viral burden in the viscera, despite robust protection in the context of overall survival. In contrast, ribavirin was able to partially knock down titers in serum, liver, and lung, but not spleen.

### Therapeutic efficacy of MY-24 in TCRV-challenged AG129 mice

**Figure 7:**
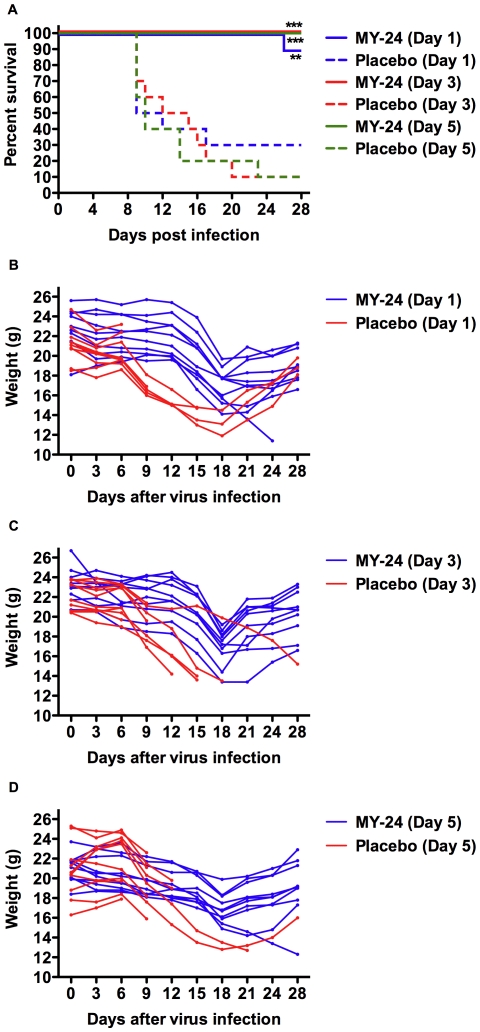
Post-exposure MY-24 treatment prevents mortality in TCRV-challenged AG129 mice and delays clinical signs of illness. Mice (n = 10/group) were treated i.p. once daily for 7 days with 75 mg/kg/day of MY-24 or placebo starting at the indicated days post infection. A) Survival and a longitudinal analysis of B) body weight were monitored during the course of the infection. Individual animal weights were recorded every 3 days for 24 days, with a final weight taken at the conclusion of the experiment. **P<0.01, ***P<0.001 compared to the respective saline placebo-treated animals.

In the first two experiments, MY-24 treatment was initiated 4 h prior to TCRV challenge. A third experiment was conducted to investigate the therapeutic capacity of MY-24. Because TCRV infection can spread into the brain by day 8 (and possibly day 7) and because we did not have any information as to the ability of MY-24 to cross the blood-brain barrier, therapeutic interventions were started prior to day 6. As seen in Figure 7A, delayed treatment of TCRV infection in AG129 mice was highly effective with complete protection afforded when treatment was initiated on or after day 3, and 90% protection in the group where treatment started on day 1. Based on individual animal weights tracked during the course of the experiment, mice receiving placebo generally began to markedly lose weight during the transition from day-6 to day-9, and the surviving mice started their recovery after day 18 (Figure 7B–D). In contrast, the MY-24 day-1 and day-3 treatment groups maintained weight through 12 days, lost considerable weight on days 15 and 18, followed by recovery and weight gain by day 21 (Figure 7B, C). Interestingly, most of the mice in the day-5 treatment initiation group were found to have a more gradual decrease in weight through day 18, prior to the recovery phase (Figure 7D).

We also documented ruffling of fur during the course of the experiment. For the placebo-treated animals, this process began on about day 7 or 8 and continued throughout the entire experiment for the surviving animals. In the day-1 MY-24 group, the ruffled appearance started on day 12 and persisted for most of the animals through the end of the study. The day-3 group began to show the ruffled appearance on day 14, but it was noted that by day 22 the ruffling was less pronounced and improved gradually afterwards. For the day-5 MY-24 treatment group, mild ruffling was seen on day 16 of infection and persisted until day 21 with minimal ruffling noted by day 22. These observations are consistent with the individual weight change profiles shown in Figure 7B–D. Remarkably, both the weight change data and our observations of the mice show that the later the time of initiation, the more efficacious the MY-24 treatment was.

### Evaluation of MY-24 in hamsters challenged with PICV

**Figure 8:**
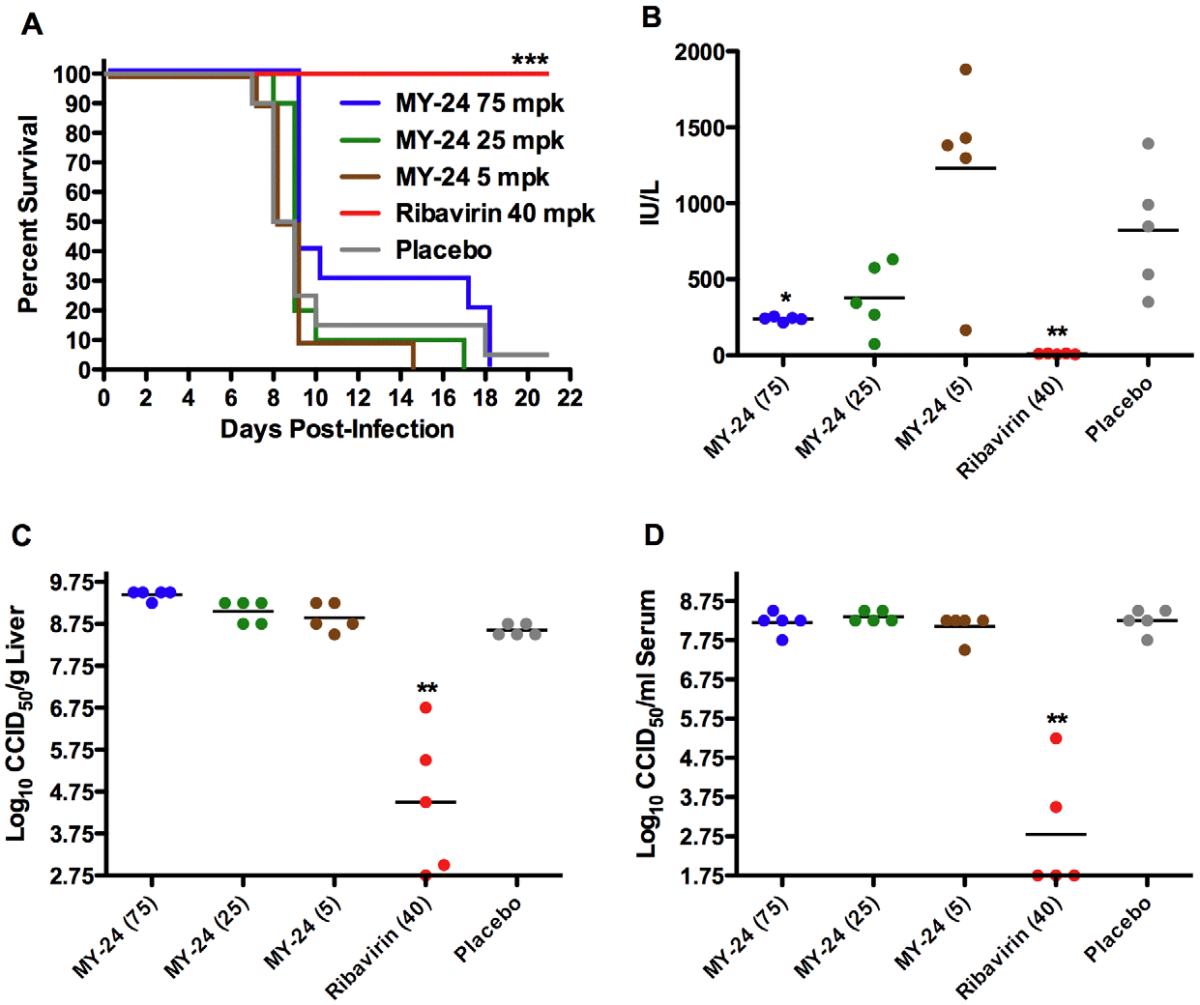
Effect of MY-24 treatment on survival outcome, viral burden, and liver disease in PICV-infected hamsters. Animals in each group (n = 15) were treated i.p. twice daily for 6 days with MY-24 or ribavirin at the indicated mg/kg/day dose levels. A placebo-treated control group (n = 25) was included for comparison. Treatment was initiated 4 hours prior to infection and 5 animals per group were sacrificed on day 7 for evaluation of viral burden and ALT levels. A) Survival analysis, B) serum ALT, C) liver virus titer, and D) serum virus titer. *P<0.05, **P<0.01, ***P<0.001 compared to saline placebo-treated animals.

We also investigated the activity of MY-24 in the well-established hamster model of acute arenaviral disease based on challenge with PICV. MY-24 was well-tolerated in preliminary toxicity studies with doses tested up to 100 mg/kg/day (data not shown). Doses ranging from 5 to 75 mg/kg/day were evaluated in the in the first challenge efficacy experiment. As seen in Figure 8A, there was a slight protective effect evident by the survival curve comparison of the high-dose MY-24 treatment with the placebo. This was emphasized by a significant increase in the mean day of death (*P*<0.05) of hamsters treated with 75 mg/kg/day (11.7±4.1 days), compared to the placebo group (9.4±3.1). The 25- and 5-mg/kg/day doses were ineffective. A MY-24 dose-dependent decrease in ALT was noted with the animals that received the 75-mg/kg/day dose presenting with significantly reduced levels (Figure 8B). Consistent with the lack of a direct effect on viral titers seen in the TCRV AG129 mouse infection model (Figure 6B–D), neither liver nor serum PICV burden was reduced in the MY-24-treated hamsters compared to animals receiving saline placebo (Figure 8C, D). The positive control drug, ribavirin, protected 100% of challenged hamsters from death and reduced viral burden by an average of 4–5.5 log_10_, and greatly minimized liver disease as reflected by baseline ALT readings (Figure 8).

Because we observed the most robust protection at the highest tested dose of 75 mg/kg/day, we also tested a higher dose of MY-24. Notably, we used smaller hamsters in the subsequent studies to reduce the MY-24 quantities needed for higher and extended 7-day duration dosing regimens.

As in the first efficacy study, 75-mg/kg/day of MY-24 had only a subtle protective effect primarily manifested as a delay in mean day of death (10.6±2.3 days compared to 8.9±1.3 days for the placebo: *P*<0.01) and significantly reduced liver disease as measured by systemic ALT concentrations (333±124 IU/ml compared to 1295±483 IU/ml for the placebo: *P*<0.001). Moreover, no reduction in serum or liver viral load was observed following MY-24 treatment (data not shown). At a dose of 100 mg/kg/day, MY-24 again had no impact on the total number of surviving hamsters, but similarly delayed the time of death (13.4±4.6 days compared to 9.7±2.7 days for the placebo: *P*<0.05). Taken together, increasing of MY-24 dosage and extending treatment duration did not remarkably improve disease outcome.

## Discussion

MY-24 is an analog of aristeromycin, which is a potent inhibitor of S-adenosyl-L-homocysteine (AdoHcy, SAH) hydrolase. SAH hydrolase was first identified as an antiviral target in 1982 [36]. Since that time, a number of compounds, including aristeromycin, have been reported to have broad-spectrum activity versus a number of viruses, including arenaviruses [28]. In the present study, we have evaluated MY-24 in two small animal models of acute arenaviral infection based on activity of the compound in cell culture.

Despite only observing a slight protective effect in trials employing the hamster clade A PICV infection model, MY-24 was highly efficacious in the newly developed AG129 mouse TCRV challenge model. Because of the better cell culture activity profile of MY-24 against the clade B arenavriuses, it was essential to test the compound against TCRV. A recent study underscores the need to test promising compounds targeting the clade B arenaviral hemorrhagic viruses against TCRV *in vivo*. Bolken *et al.* used a model based on i.p. challenge of in newborn mice [6] to demonstrate the efficacy of a small molecule inhibitor (ST-294) with known *in vitro* activity against clade B NWA, but not the more distantly related PICV or LASV [4].

Because rodent model systems that employ newborn animals to produce lethal infection are generally considered to be farther removed from the human disease being modeled [3], and because of the difficulty of working with newborn mice, we developed a new TCRV mouse infection model based on i.p. challenge of AG129 type I and type II IFN receptor-deficient mice. The use of the new TCRV mouse model over the existing hamster PICV model was also advantageous because it greatly reduced the amount of compound required for our studies. On a mg/kg basis, a TCRV mouse experiment would require ∼5- to 8-fold less drug to complete compared to hamster models. This can be an important issue in early stage drug development wherein costly, time-consuming synthesis of additional compound would be pursued only if dictated by basic proof-of-concept studies in mice, prior to advancing to PICV, Pirital virus [37], or BSL-4 arenaviral hemorrhagic fever guinea pig models [38], [39], [40]. One must consider however that the use of the new TCRV model is probably better suited for investigating the activity of candidate therapeutics that directly target the virus life-cycle and/or do not require complete host IFN pathways to impart their antiviral activity.

Despite the inhibition of arenavirus replication in cell culture model systems, MY-24 did not reduce the viral burden systemically nor in the various hamster or mouse tissues examined. This may suggest that the mode of action *in vivo* is not based on SAH hydrolase or other virus-direct antiviral activity. However, we cannot rule out the possibility that at earlier time points, we may have observed a significant reduction in serum and/or tissue viral burden. It is conceivable that viral replication is abrogated at earlier stages of the infection, yet eventually the titers reach peak levels at the time of our analysis just prior to when the placebo-treated mice begin to succumb to the disease. Additional sampling times shortly after the onset of MY-24 treatment may uncover differences in viral replication kinetics early during the course of infection. Nevertheless, the present findings are consistent with the idea that MY-24 may ameliorate disease by blunting host response factors that play a role in disease pathogenesis occurring in response to the viral infection.

In PICV-challenged hamsters, MY-24 significantly reduced liver disease as indicated by greatly reduced levels of ALT. In the AG129 mice treated therapeutically, the data are consistent with a delay in disease pathogenesis as weight loss and visual signs of disease were delayed in animals that received MY-24. This and the hamster ALT findings support the theory that the compound is attenuating the development of disease. A delay in the progression of disease may facilitate the eventual clearance of the virus from the system once the humoral immune response has more fully developed up in the TCRV-infected mice. In hamsters, the delay in disease pathogenesis does not lead to improved survival, only a delay in time of death. Presumably, the hypercytokinemia that has developed in response to the PICV infection in immune competent animals ultimately triggers excessive vascular leakage (Gowen *et al.* unpublished data), believed to be the fatal lesion in cases of VHF [41], [42].

Because our present studies only examined viral titers on day 8 of infection, studies to evaluate viral titers on day 14, 21 and 28 of infection in MY-24-treated mice are necessary to determine when the virus is cleared from the blood and tissues, and to assess anti-TCRV neutralizing antibody levels. Moreover, future studies will examine the effect of MY-24 treatment on aspartate aminotransferase (AST) levels in TCRV-infected mice. Elevation of systemic concentration of AST is indicative of tissue damage and is a known prognosticator of disease outcome in severe cases of Lassa fever [14]. Lastly, our findings showed that in mice wherein treatment was initiated on day 5 of infection, disease was less severe and the onset of clinical signs of illness was delayed. This may be due to MY-24 possibly dampening the exaggerated cytokine response that may occur; however, starting treatment too soon may prevent a robust early innate immune response. Thus, additional experiments to investigate the contribution of an overzealous proinflammatory response to mortality in the AG129 TCRV mouse infection model, and the impact of MY-24 treatment on this response, are warranted. These studies will hopefully provide insights into the mechanism by which mice treated with MY-24 are able to survive TCRV challenge.

## Supporting Information

Figure S1 (0.20 MB DOC)

Text S1 (0.04 MB DOC)
