# Supplementary material for: Development of a New Tacaribe Arenavirus Infection Model and Its Use to Explore Antiviral Activity of a Novel Aristeromycin Analog
Source: PLoS One. 2010 Sep 16;5(9):e12760. doi: 10.1371/journal.pone.0012760 (PMC2940843; doi:10.1371/journal.pone.0012760)
Supplement: Figure S1 — (0.20 MB DOC) [file pone.0012760.s001.doc]

Reagents and conditions: (a) (i) Ethyl (trimethylsilyl)acetate, n-BuLi, DIPA, HMPA/THF, -78 ˚C, (ii) KF, EtOH/H_2_O, rt, 84%; (b) NaBH_4_, 100%; (c) PPh_3_, DIAD, 6-chloropurine, THF, 0-50 ˚C, 52%; (d) DIBAL, DCM, -50 ˚C, 80%; (e) NH_3_/MeOH, 120 ˚C, 94%; (f) 1N HCl/MeOH, 95% [34].
