# Supplementary material for: Development of a New Tacaribe Arenavirus Infection Model and Its Use to Explore Antiviral Activity of a Novel Aristeromycin Analog
Source: PLoS One. 2010 Sep 16;5(9):e12760. doi: 10.1371/journal.pone.0012760 (PMC2940843; doi:10.1371/journal.pone.0012760)
Supplement: Text S1 — (0.04 MB DOC) [file pone.0012760.s002.doc]

*Synthesis of MY-24*

All melting points were recorded on a Meltemp II melting point apparatus and the values were uncorrected. The combustion analysis was performed at Atlantic Microlab, Norcross, GA. ^1^H and ^13^C NMR spectra were recorded either on Bruker AC 250 spectrometer (operated at 250 and 62.9 MHz, respectively) or Bruker AC 400 spectrometer (operated at 400 and 100 MHz, respectively). All referenced to internal tetramethylsilane (TMS) at 0.0 ppm. Reactions were monitored by thin-layer chromatography (TLC) using 0.25 mm Whatman Diamond silica gel 60-F_254_ precoated plates with visualization by irradiation with a Mineralight UVGL-25 lamp. Column chromatography was performed on Whatman silica, 230-400 mesh, 60 Å and elution with the indicated solvent system. Yields refer to chromatographically and spectroscopically (^1^H and ^13^C NMR) homogeneous materials.

**Ethyl 2-[(3a*R*, 4*R*, 6a*R*)-2,2-Dimethyl-6-oxo-tetrahydro-cyclopenta[1,3]dioxol-4-yl]acetate (5)**: n-Butyllithium (12.0 mL, 2.5 M in hexane, 30 mmol) was added dropwise in 5 min to a cooled (~ -5 °C) solution of di*iso*propylamine (4.0 mL, 28 mmol) in THF (80 mL). After stirring for 10 min at this temperature, this solution was cooled further to -40 °C. Ethyl trimethylsilylacetae (4.0 mL, 22 mmol) was then added dropwise in 10 min. The resulting solution was kept at this temperature for 40 min followed by addition of HMPA/THF (24.0 mL, 1:1). The solution was cooled further to -78 °C and **4** (3.08 g, 20.0 mmol) in THF (10 mL) was added dropwise. The reaction mixture was kept stirring at this temperature for 1.5 h and then allowed to gradually rise to -40 °C. Saturated ammonium chloride (40 mL) was added at this point. After the reaction was warmed to room temperature and stirred at this temperature for 1 h, the organic layer was separated and aqueous layer was extracted with CH_2_Cl_2_ (3 x 100 mL). The combined organic phases were evaporated. To the residue was added a solution of KF (1.50 g, 25.9 mmol) in EtOH/H_2_O (2 : 1) (40 mL). Reaction was stirred at room temperature for 6 h. The mixture was extracted with CH_2_Cl_2_ (4 x 100 mL). The combined organic phases were dried (anhydrous Na_2_SO_4_). Evaporation and column chromatography (EtOAc/hexanes = 1:3) afforded **5** as a pale yellow liquid (4.05 g, 84 %). ^1^H NMR (250 MHz, CDCl_3_): 4.60 (d, J = 5.5 Hz, 1H), 4.38 (d, J = 5.5 Hz, 1H), 4.14 (q, J = 7.0 Hz, 2H), 2.91-2.74 (m, 2H), 2.51-2.48 (m, 2H), 2.16-2.09 (m, 1H), 1.44 (s, 3H), 1.34 (s, 3H), 1.26 (t, J = 7.0 Hz, 3H). ^13^C NMR (62.9 MHz, CDCl_3_): 212.9, 171.6, 112.0, 82.0, 78.7, 60.9, 39.6, 37.6, 33.8, 26.8, 24.7, 14.1. Anal. Calcd for C_12_H_18_O_5_: C, 59.49; H, 7.49. Found: C, 59.50; H, 7.48.

**Ethyl 2-[(3a*R*,4*R*,6*S*,6a*S*)- 2,2-Dimethyl-6-hydroxy-tetrahydro-cyclopenta[1,3]dioxol-4-yl]acetate (6)**. Sodium borohydride (150 mg, 3.95 mmol) was added in portions to an ice-cold solution of **5** (0.64 g, 2.6 mmol) in MeOH (15 mL). The reaction mixture was stirred at this temperature for 1.5 h. Saturated NH_4_Cl solution (4.0 mL) was added. The mixture was extracted with CH_2_Cl_2_ (3 x 20 mL). The combined organic phases were washed with brine and dried (anhydrous Na_2_SO_4_). Evaporation of the solvent afforded **6** as a colorless oil (0.65 g, 100%). ^1^H NMR (250 MHz, CDCl_3_): 4.51 (dd, J = 5.7 Hz, 1H), 4.39 (dd, J = 6.0, 1.4 Hz, 1H), 4.13 (q, J = 7.2 Hz, 2H), 4.08 (br, 1H), 2.52 (m, 1H), 2.35-2.24 (m, 2H), 1.95 (m, 1H), 1.74 (m, 1H), 1.51(s, 3H), 1.34 (s, 3H), 1.30 (t, J = 7.2 Hz, 3H). ^13^C NMR (62.9 MHz, CDCl_3_): 172.0, 111.9, 84.3, 79.3, 71.2, 60.7, 38.2, 37.0, 36.7, 26.2, 24.4, 14.3. Anal. Calcd for C_12_H_20_O_5_: C, 59.00); H, 8.25. Found: C, 59.26; H, 8.32.

**Ethyl 2-[(3a*R*,4*R*,6*S*,6a*S*)- 2,2-Dimethyl-6-(6-chloropurin-9-yl)-tetrahydro-cyclopenta[1,3]dioxol-4-yl]acetate (7)**. D*iis*opropyl azodicarboxylate (3.96 g, 19.6 mmol) in THF (10 mL) was added dropwise to an ice-bath chilled suspension of **6** (3.20 g, 13.1 mmol), 6-chloropurine (2.66 g, 17.0 mmol) and Ph_3_P (5.15 g, 19.6 mmol) in THF (100 mL). A white complex formed in 5 min. The resulting mixture was stirred at the same temperature for 30 min then was brought to 50 °C for 48 h. Evaporation and purification by column chromatography (EtOAc/hexanes = 1:2) afforded **7** as viscous foam (2.50 g, 52 %). ^1^H NMR (250 MHz, CDCl_3_): 8.74 (s, 1H), 8.28 (s, 1H), 5.13 (m, 1H), 4.90 (m, 1H), 4.65 (m, 1H), 4.19 (q, J = 7.2 Hz, 2H), 2.80-2.43 (m, 5H), 1.59 (s, 3H), 1.32 (s, 3H), 1.27 (t, J = 7.2 Hz, 3H). ^13^C NMR (62.9 MHz, CDCl_3_): 171.7, 151.6, 151.2, 144.8, 132.3, 114.2, 83.4, 83.3, 62.1, 60.7, 60.3, 40.3, 37.2, 36.3, 27.5, 25.2, 14.2. Anal. Calcd for C_17_H_21_N_4_O_4_: C, 53.62; H, 5.52; N, 14.72; Cl, 9.31. Found: C, 53.58; H, 5.71; N, 14.43; Cl, 9.05.

**2-[(3a*R*,4*R*,6*S*,6a*S*)-6-(6-Chloro-purin-9-yl)-2,2-dimethyl-tetrahydro-cyclopenta[1,3]dioxol-4-yl]-ethanol (8)**. Di*iso*butylaluminum hydride (4.5 mL, 1.0 M in CH_2_Cl_2_, 4.5 mmol) was added dropwise to a solution of **7** (0.35 g, 0.92 mmol) in CH_2_Cl_2_ (15 mL) at -50 °C. The reaction mixture was stirred at this temperature for 3 h before being quenched by adding MeOH (2 mL) and H_2_O (10 mL). After the reaction mixture was warmed to room temperature, the organic layer was separated and the aqueous layer was extracted with CH_2_Cl_2_ (4 x 10 mL). The combined organic phases were dried (anhydrous Na_2_SO_4_). After the removal of the solvent, the residue was purified by column chromatography (EtOAc) to afford **8** as a white foam (0.25 g, 80 %). ^1^H NMR (250 MHz, CDCl_3_): 8.75 (s, 1H), 8.23 (s, 1H), 5.10 (m, 1H), 4.88 (m, 1H), 4.62 (m, 1H), 3.82 (m, 2H), 2.65 (br, 1H), 2.57-2.32 (m, 3H), 1.87 (m, 2H), 1.58 (s, 3H), 1.32 (s, 3H). ^13^C NMR (62.9 MHz, CDCl_3_): 151.8, 151.3, 144.9, 132.4, 114.4, 84.4, 83.6, 62.2, 61.3, 42.0, 37.2, 35.9, 27.5, 25.2. Anal. Calcd for C_15_H_19_ClN_4_O_3_: C, 53.18; H, 5.65; N, 16.54. Found: C, 53.28; H, 5.84; N, 16.93.

**2-[(3a*R*,4*R*,6*S*,6a*S*)-6-(6-amino-purin-9-yl)-2,2-dimethyl-tetrahydro-cyclopenta[1,3]dioxol-4-yl]-ethanol (9)**. Compound **8** (250 mg, 0.78 mmol) was dissolved in saturated methanolic ammonia solution (10 mL) (extra space) in a stainless steel pressure vessel and then heated at 120 °C for 48 h. After cooling to 0 °C, the reaction vessel was opened and the ammonia and MeOH allowed to evaporate to dryness. Column chromatography (EtOAc/MeOH = 7:1) of the residue afforded **9** as a white solid (221 mg, 94 %), mp: >160 °C (dec). ^1^H NMR (250 MHz, CDCl_3_): 8.28 (s, 1H), 8.14 (s, 1H), 7.25 (s, 2H), 5.00 (m, 1H), 4.77 (m, 1H), 4.44 (m, 2H), 3.45 (m, 2H), 2.31 (m, 1H), 2.11 (m, 2H), 1.71 (m, 1H), 1.56 (m, 1H), 1.46 (s, 3H), 1.22 (s, 3H). ^13^C NMR (62.9 MHz, CDCl_3_): 156.1, 152.4, 149.4, 139.9, 119.2, 112.8, 84.2, 83.2, 60.1, 59.2, 42.8 36.9, 36.4, 27.4, 25.1. Anal. Calcd for C_15_H_21_N_5_O_3_·0.3 MeOH: C, 55.86; H, 6.80; N, 21.29. Found: C, 55.81; H, 6.75; N, 21.23.

**(1*R*, 2*S*, 3*R*, 5*R*)-3-(6-Amino-purin-9-yl)-5-(2-hydroxyethyl)-cyclopentane-1,2-diol (3)**. Compound **9** (210 mg, 0.66 mmol) was added to a solution of 1N HCl (8 mL) in MeOH (8 mL). The reaction mixture was stirred at room temperature for 3 h. The solvent was removed under vaccum and the residue was redissolved in distilled H_2_O (5 mL) and MeOH (5 mL). Solution was then treated with weakly basic exchange resin (Amberlite IRA-67). Filtration and evaporation afforded **3** as white solid (175 mg, 95%), mp: 178-179 °C. ^1^H NMR (400 MHz, DMSO-d_6_): 8.20 (s, 1H), 8.11 (s, 1H), 7.17 (s, 2H), 4.90 (d, J = 6.27 Hz, 1H), 4.68 (d, J = 4.63 Hz, 1H), 4.57 (m, 1H), 4.43 (t, J = 5.12, 1H), 4.35 (m, 1H), 3.71 (m, 1H), 3.46 (m, 2H), 2.24 (m, 1H), 1.97 (m, 1H), 1.74 (m, 2H), 1.58 (m, 1H), ^13^C NMR (100 MHz, DMSO): 156.0, 152.1, 149.6, 140.2, 119.3, 74.9, 74.3, 59.9, 59.4, 40.0, 37.4, 32.5. Anal. Calcd for C_12_H_17_N_5_O_3_: C, 51.60; H, 6.14; N, 25.08. Found: C, 51.39; H, 6.18; N, 24.81.
